# Identification of a prognostic gene signature of colon cancer using integrated bioinformatics analysis

**DOI:** 10.1186/s12957-020-02116-y

**Published:** 2021-01-13

**Authors:** Zhengyu Fang, Sumei Xu, Yiwen Xie, Wenxi Yan

**Affiliations:** 1 Department of Anorectal Surgery, The First Affiliated Hospital of Zhejiang Chinese Medical University, Hangzhou, 310006 Zhejiang Province China; 2 Department of General Practice, The First Affiliated Hospital of Zhejiang Chinese Medical University, #54 Youdian Road, Shangcheng District, Hangzhou, 310006 Zhejiang Province China; 3 Department of Clinical Laboratory, The First Affiliated Hospital of Zhejiang Chinese Medical University, Hangzhou, 310006 Zhejiang Province China

**Keywords:** Weighted gene co-expression network analysis, Meta-analysis, Prognostic model, Overall survival, Colon cancer

## Abstract

**Background:**

Colon cancer is a worldwide leading cause of cancer-related mortality, and the prognosis of colon cancer is still needed to be improved. This study aimed to construct a prognostic model for predicting the prognosis of colon cancer.

**Methods:**

The gene expression profile data of colon cancer were obtained from the TCGA, GSE44861, and GSE44076 datasets. The WGCNA module genes and common differentially expressed genes (DEGs) were used to screen out the prognosis-associated DEGs, which were used to construct a prognostic model. The performance of the prognostic model was assessed and validated in the TCGA training and microarray validation sets (GSE38832 and GSE17538). At last, the model and prognosis-associated clinical factors were used for the construction of the nomogram.

**Results:**

Five colon cancer-related WGCNA modules (including 1160 genes) and 1153 DEGs between tumor and normal tissues were identified, inclusive of 556 overlapping DEGs. Stepwise Cox regression analyses identified there were 14 prognosis-associated DEGs, of which 12 DEGs were included in the optimized prognostic gene signature. This prognostic model presented a high forecast ability for the prognosis of colon cancer both in the TCGA training dataset and the validation datasets (GSE38832 and GSE17538; AUC > 0.8). In addition, patients’ age, T classification, recurrence status, and prognostic risk score were associated with the prognosis of TCGA patients with colon cancer. The nomogram was constructed using the above factors, and the predictive 3- and 5-year survival probabilities had high compliance with the actual survival proportions.

**Conclusions:**

The 12-gene signature prognostic model had a high predictive ability for the prognosis of colon cancer.

**Supplementary Information:**

The online version contains supplementary material available at 10.1186/s12957-020-02116-y.

## Introduction

As one of the most common gastrointestinal malignant diseases, colon cancer is a worldwide leading cause of cancer-related mortality [1, 2]. Of the 36 cancers estimated globally in 2018, the number of new cases and related deaths of colon cancer ranked fourth, with estimated new cases of approximately 1,100,000 [2]. The current standard therapeutic strategy for colon cancer is the combination of surgery and adjuvant chemotherapy or radiation therapy [3]. However, the prognosis of patients with colon cancer varies by multiple factors, including the clinical histological subtypes, age, genetic profiles, and treatment responses [4–8]. Also, the unsatisfactory prognostic outcomes still exist due to the complex pathogenesis that involves a variety of molecular or genetic factors [3, 9–12]. Therefore, the identification of prognostic biomarkers for colon cancer is still necessary.

The advances of biomarkers identified by high-throughput genome sequencing and bioinformatics analysis have attracted a great amount of interest in the last two decades. Computational bioinformatics analysis identifies potential biomarkers by deducing the association with disease status and progression. Most important of all, some of them are verifiable and reliable in clinical trials [13, 14]. For instance, Dalerba et al. [15] emphasized that the lack of the caudal-related homoeobox transcription factor 2 (CDX2) is associated with a poor prognosis in patients with stage II/III colon cancers using bioinformatics analysis. Besides, the association between the loss of CDX2 expression and poor disease-free survival in two Denmark cohorts of patients with colon cancer was validated by Hansen et al. [13]. These results showed that computational bioinformatics tools are of great value for identifying and providing potential prognostic biomarkers before the implements of clinical or preclinical experiments.

In the past decades, a lot of data mining analysis of mRNA, microRNA, long non-coding RNA, and DNA methylation have been performed on human cancers, including colon cancer [16–19]. As the biomarkers identified by the above techniques are of diagnostic and prognostic values in cancers and the revolution of sequencing technologies and bioinformatics tools facilitates the identification of more potential biomarkers related to disease progression [20–23], the more potential biomarkers identified, the more recognition and options for the diagnosis and treatment of colon cancer.

This current study aimed to identify a potential prognostic biomarker or gene signature using bioinformatics analysis. An integrated bioinformatics analysis was performed using The Cancer Genome Atlas (TCGA) and microarray datasets in the gene expression omnibus (GEO) database. The differentially expressed genes (DEGs) between the colon tumor and non-tumor control tissues and prognosis-associated genes were identified and used for the construction of a gene signature with prognostic predictive power. The possibility of using the prognostic model as a biomarker for colon cancer was validated using different cohorts. This study may provide a clinical reference for predicting the survival probability of patients with different clinical subtypes.

## Materials and methods

### Data extraction

The public colon cancer gene expression profiles data were preliminarily extracted from the National Center for Biotechnology Information (NCBI) GEO repository (https://www.ncbi.nlm.nih.gov/geo/) using the search words “colon cancer”. Datasets selected if they met the following inclusion criteria: (1) human gene expression profiles data, and (2) inclusive of ≥ 100 tissue samples, with or without control samples; and (3) for datasets without control samples, the clinical prognosis information of the tumor samples were included. Four datasets were selected according to the above criteria, including GSE44861 (Affymetrix-GPL3921 [HT_HG-U133A] platform, 56 tumor samples and 55 normal samples), GSE44076 (Affymetrix-GPL13667 [HG-U219] platform, 98 tumor samples and 148 normal samples), GSE17538 (Affymetrix-GPL570 [HG-U133_Plus_2] platform, 238 tumor samples), and GSE38832 (Affymetrix-GPL570 [HG-U133_Plus_2] platform, 122 tumor samples). The first two datasets with control samples were for the identification of DEGs using the weighted gene co-expression network analysis (WGCNA) and MetaDE analysis. The last two datasets with the clinical stage and survival data and without control samples were used for the construction of the prognostic prediction model.

**Fig. 1:**
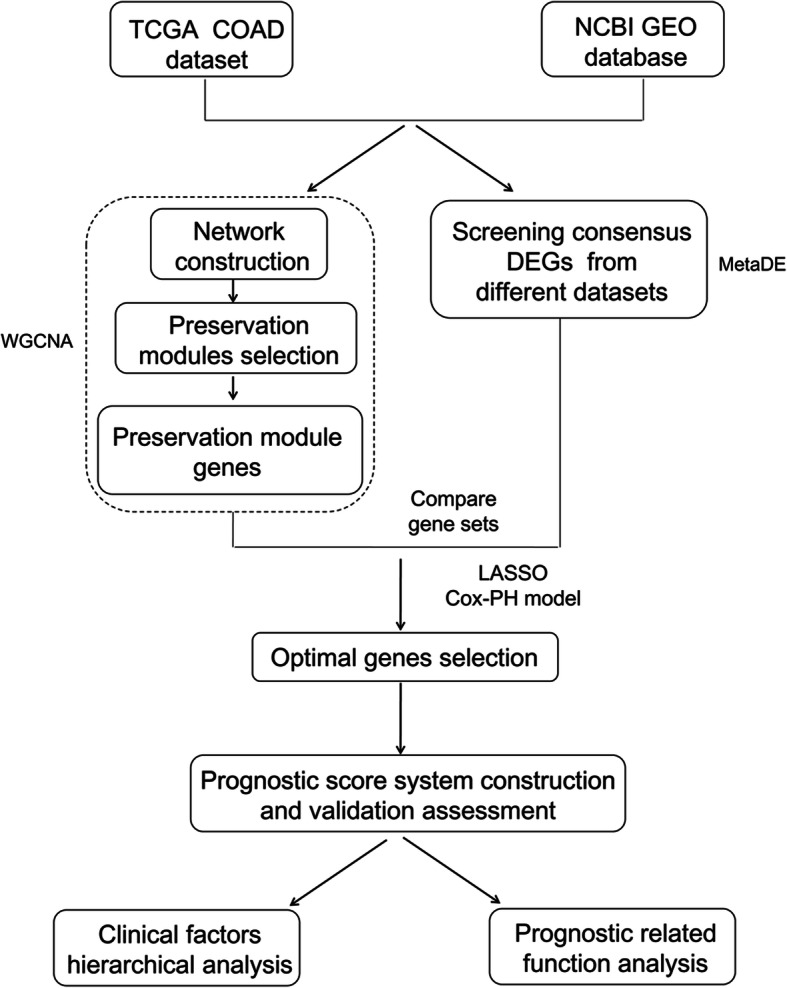
Workflow of this study. COAD, colon adenocarcinoma. DEG, differentially expressed genes. WGCNA, weighted gene co-expression network analysis. TCGA, The Cancer Genome Atlas. NCBI, National Center for Biotechnology Information. GEO, gene expression omnibus

Besides, the RNA-seq data of colon cancer and the corresponding clinical information were downloaded from TCGA (https://gdc-portal.nci.nih.gov/). After sample selection, 473 samples including 432 tumor samples with clinical information and 41 normal samples were retained in this study. A workflow of this study is shown in Fig. 1.

### Screening of colon cancer-related gene module

WGCNA has been widely applied to identify the gene module associated with diseases and extract potential therapeutic targets [24]. WGCNA software (version 1.61; https://cran.r-project.org/web/packages/WGCNA/index.html) [25] in R3.4.1 was used to screen the colon cancer-related stable gene modules with the following criteria: min size ≥ 150 and cutHeight = 0.99. The TCGA data were utilized as the training set, and the GSE44861 and GSE44076 datasets were used as the validation sets for the identification of stable gene co-expression modules. The preservation and correlation properties of the above WGCNA modules were analyzed, and modules with a preservation *Z*-score of > 5.0 and correlation *p* value of < 0.05 were defined as colon cancer-related stable gene modules.

### DEG identification by meta-analysis

The common DEGs across the TCGA, GSE44861, and GSE44076 datasets were identified using the MetaDE.ES methods in the R MetaDE package (https://cran.r-project.org/web/packages/MetaDE/) [26, 27]. Briefly, the heterogeneity test of gene expression profiles from different platforms was first conducted according to the statistical tau2, Q value, and Q pval. The common DEGs were screened out according to the following criteria: tau^2^ = 0, *p* < 0.05, Q pval > 0.05, false discovery rate (FDR) < 0.05, and log_2_fold change (FC) had the same differential expression direction across the three datasets (> 0 or < 0). The overlapping genes between the above WGCNA module genes and the common DEGs across the three datasets were retained and used for further functional enrichment analysis and the construction of the prognostic prediction model.

### Functional enrichment analysis

To investigate the biological functions associated with the above overlapping genes (DEGs), functional enrichment analyses were performed. The Gene Ontology biological processes and Kyoto Encyclopedia of Genes and Genomes (KEGG) pathways associated with these DEGs were identified using the DAVID online tool (version 6.8; https://david.ncifcrf.gov/) [28, 29]. Significant enrichment was considered when *p* value < 0.05.

### Construction and evaluation of prognostic prediction model

Before the construction of the prognostic prediction model, the prognosis-associated DEGs were identified using the univariate and multivariate Cox regression analysis in the R survival package (version 2.4, https://cran.r-project.org/web/packages/survival/index.html) [30]. The prognosis-associated DEGs in the TCGA training set (*n* = 432) were identified when log-rank *p* value < 0.05. Then, the optimal prognostic gene signature was identified using the L1-penalized least absolute shrinkage and selection operator (LASSO) Cox-proportional hazards (Cox-PH) model (lamba = 1000) in the penalized package (version 0.9-50, http://bioconductor.org/packages/penalized/) [31, 32]. Subsequently, the prognosis risk score of each sample was calculated using the following gene signature model: risk score = ∑β_gene_ × Exp_gene_, where *β* represents the LASSO coefficient and Exp denotes the expression level. All the samples in the TCGA training set were divided into the high- and low-risk groups according to the median risk score. The Kaplan-Meier (K-M) curve analysis in the R survival package (version 2.41-1) and the receiver operating characteristic (ROC) curve were used to assess the association of the risk score with the overall survival in patients with colon cancer. Similarly, the samples in the validation sets (GSE17538 and GSE38832) were separately divided into the high- and low-risk groups according to the above prognostic model. The performance of the above gene signature model in predicting the prognosis of colon cancer was validated in the validation sets (GSE17538 and GSE38832) using the K-M survival test and ROC curves.

### Identification of clinical factors associated with the prognosis of colon cancer

The clinical factors associated with the prognosis of colon cancer were identified in the TCGA training set using the univariate and multivariate Cox regression analysis of the survival package (version 2.41-1) in R3.4.1. The threshold was log-rank *p* value < 0.05. Also, the K-M survival test was used to validate the performance of the gene signature model in predicting the prognosis of patients with different clinical subtypes.

### Nomogram survival model analysis

The final nomogram was established using the “rms” package (Version 5.1-2; https://cran.r-project.org/web/packages/rms/index.html) in R3.4.0 to estimate the individualized survival probability for patients with colon cancer. The prognosis-associated clinical factors and the gene signature model were used for the construction of the nomogram. Each factor in the nomogram was ascribed points according to its weight. The total point of each sample was calculated and the 3- and 5-year survival probabilities of each sample were predicted accordingly.

### Screening of DEGs between the high- and low-risk groups

At last, the DEGs between the samples in the high- and low-risk groups were identified to investigate the different gene expression profiles and features between patients with different survival probabilities. The DEGs between the high- and low-risk groups in the training set were screened using the limma package (Version 3.34.7, https://bioconductor.org/packages/release/bioc/html/limma.html) [33], with the thresholds of FDR < 0.05 and |log_2_FC| > 0.5.

## Results

### Extraction of WGCNA modules related to colon cancer

**Fig. 2:**
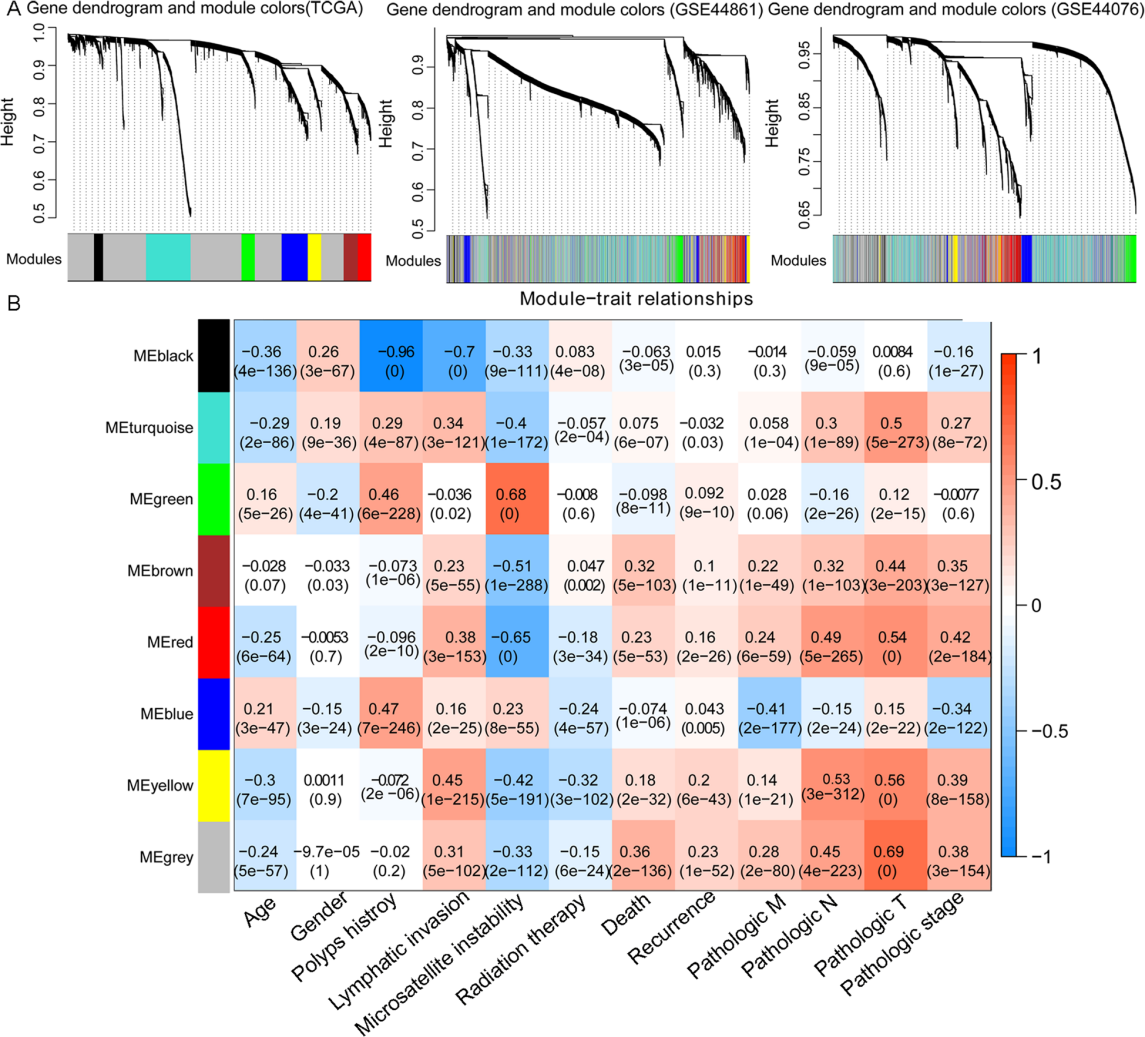
The gene module related to colon cancer based on the weighted gene co-expression network analysis (WGCNA) algorithm. **a** The module partition results of WGCNA in the TCGA (left), GSE44861 (middle), and GSE44076 (right) datasets, respectively. The different colors represent the different WGCNA modules. **b** The correlation heatmap of gene modules with the clinical factors of colon cancer. The horizontal axis represents clinical factors, and the vertical axis represents gene modules. The color changed from blue to red indicates the correlation from negative to positive. The numbers in the boxes indicate the correlation coefficients (upper) and the numbers in parentheses indicate the *p* values (lower)

The correlation analysis of RNA-seq data showed there were significant positive correlations (expression correlation coefficient > 0.700 and *p* < 1e^−200^) and connectivities (*p* < 1e^−06^) across the TCGA, GSE44861, and GSE44076 datasets (Figure S1A). Before the identification of the WGCNA modules analysis, the scale-free topology criterion was identified: the soft threshold power = 7 when the scale-free topology model fit R^2^ was maximized (R^2^ = 0.9; Figure S1B). Then, 8 WGCNA modules were identified in the training dataset according to the criteria: soft threshold power = 7, min size ≥ 150, and cutHeight = 0.99 (Fig. 2a). The same module division was identified in the two validation datasets (GSE44861 and GSE44076; Fig. 2a).

Subsequently, 5 robust modules (blue, brown, green, red, and yellow) with a preservation *Z*-score of > 5.0 and a *p* value of < 0.05 were obtained. A total of 1160 genes, including 381, 205, 195, 184, and 195 genes in the blue, brown, green, red, and yellow modules, were obtained (Table 1). The correlation of these 8 WGCNA modules with clinical factors, including patients’ age, gender, history of colon polyps, lymphatic invasion, microsatellite instability, radiation therapy, death, tumor recurrence, pathologic M, pathologic N, pathologic T, and pathologic stage, is shown in Fig. 2b. For instance, the genes in the red module were significantly correlated with the pathologic T classification (cor = 0.54, *p* < 0.0001).

**Table 1:** The weighted gene co-expression network analysis (WGCNA) gene modules related to colon cancer

ID	Color	Module size	Preservation	
			***Z***-score	***P*** value
Module 1	Black	133	1.9913	1.40E−01
Module 2	**Blue**	381	8.7017	4.50E−06
Module 3	**Brown**	205	10.4907	4.00E−03
Module 4	**Green**	195	8.2073	5.10E−03
Module 5	Grey	2469	0.3400	2.30E−05
Module 6	**Red**	184	10.9777	1.00E− 03
Module 7	Turquoise	649	4.0049	1.30E−02
Module 8	**Yellow**	195	5.6788	2.00E−05

### Identification of common DEGs using the MetaDE analysis

**Fig. 3:**
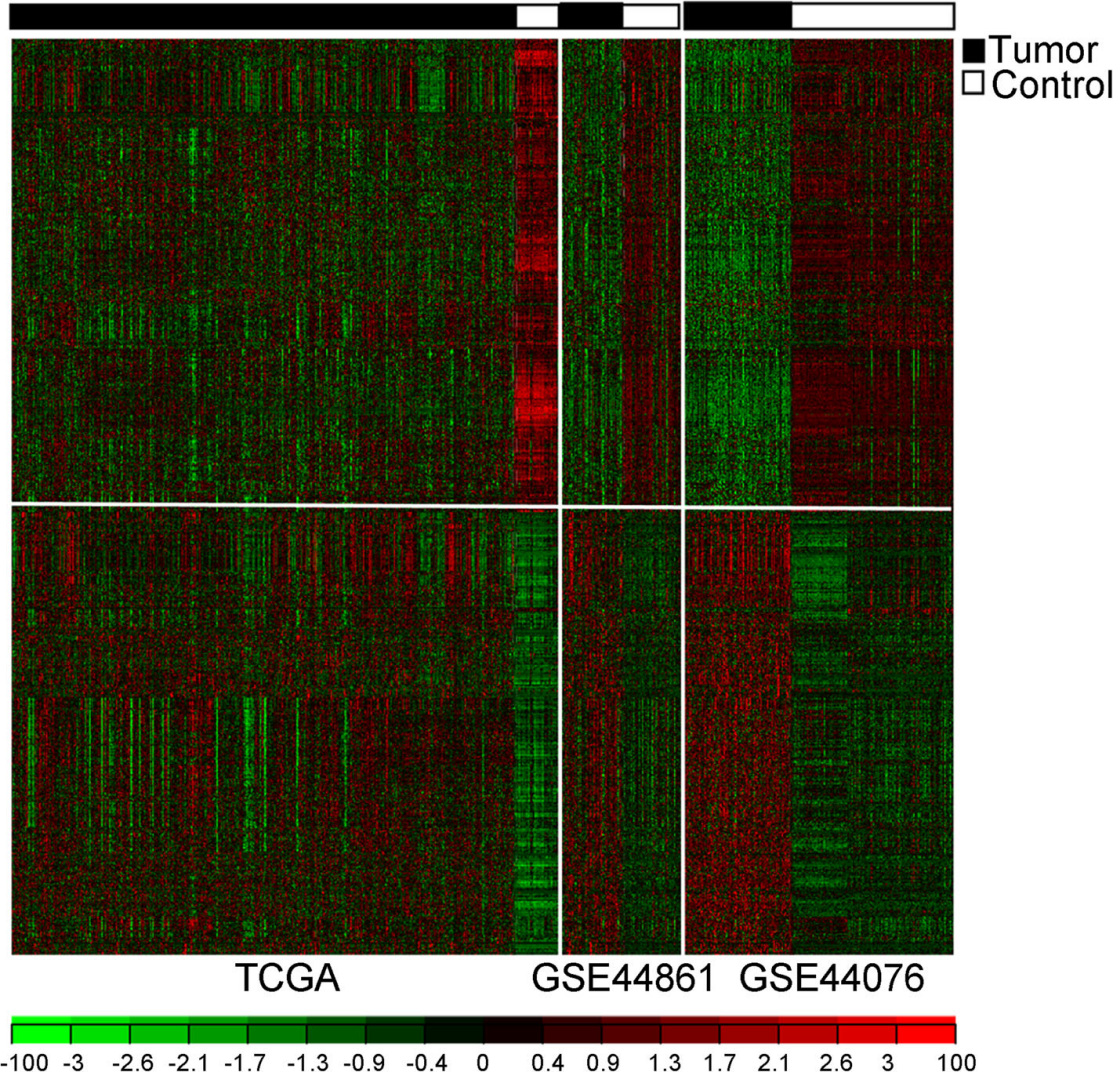
The heatmap of the common differentially expressed genes across the three datasets. High- and low-expression levels are indicated by red and green, respectively

Following the aforementioned criteria for the MetaDE analysis, 1153 common DEGs were identified across the three datasets (TCGA, GSE44861, and GSE44076), including 724 downregulated DEGs and 429 upregulated DEGs. These DEGs had distinctively different expression profiles in the tumor and control samples and showed the same differential expression direction across the three datasets (Fig. 3).

### Enrichment analysis of common DEGs

**Fig. 4:**
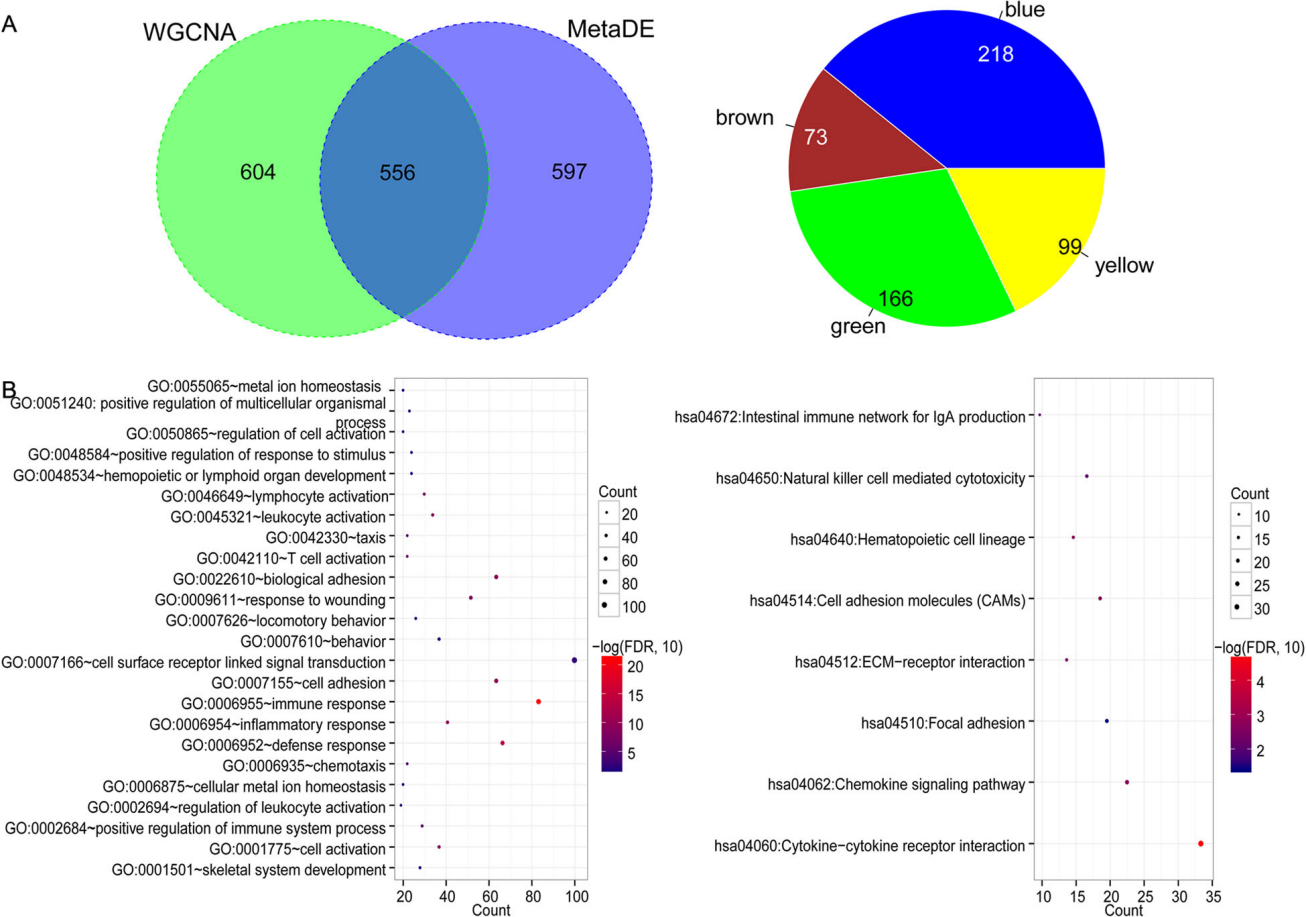
Features of the differentially expressed genes (DEGs) in the cancer-related WGCNA genes modules. **a** The Venn diagram indicating the overlapping genes between genes in the five cancer-related WGCNA modules and the common DEGs across the three datasets (TCGA, GSE44861, and GSE44076) identified by the MetaDE analysis (left), and the pie chart showing the number of overlapping genes in WGCNA modules (right). **b** The Gene Ontology biological processes (left) and Kyoto Encyclopedia of Genes and Genomes pathways (right) associated with the overlapping genes in the above figures. Horizontal axis represents gene number. The color and size of the dots indicate the *p* value. The closer the color is to red, the higher the significance

The Venn diagram indicated that 556 genes were overlapped between the five WGCNA module genes (*n* = 1160) and common DEGs (*n* = 1153) were obtained (Fig. 4a), including 218, 73, 166, 0, and 99 genes in the blue, brown, green, red, and yellow modules, respectively. The functional enrichment analyses indicated that these common DEGs were significantly associated with 24 biological processes related to immune response and the defense response (Fig. 4b) and 8 KEGG pathways including cytokine-cytokine receptor interaction, chemokine signaling pathway, and focal adhesion (Fig. 4b).

### Construction of the prognostic model

**Fig. 5:**
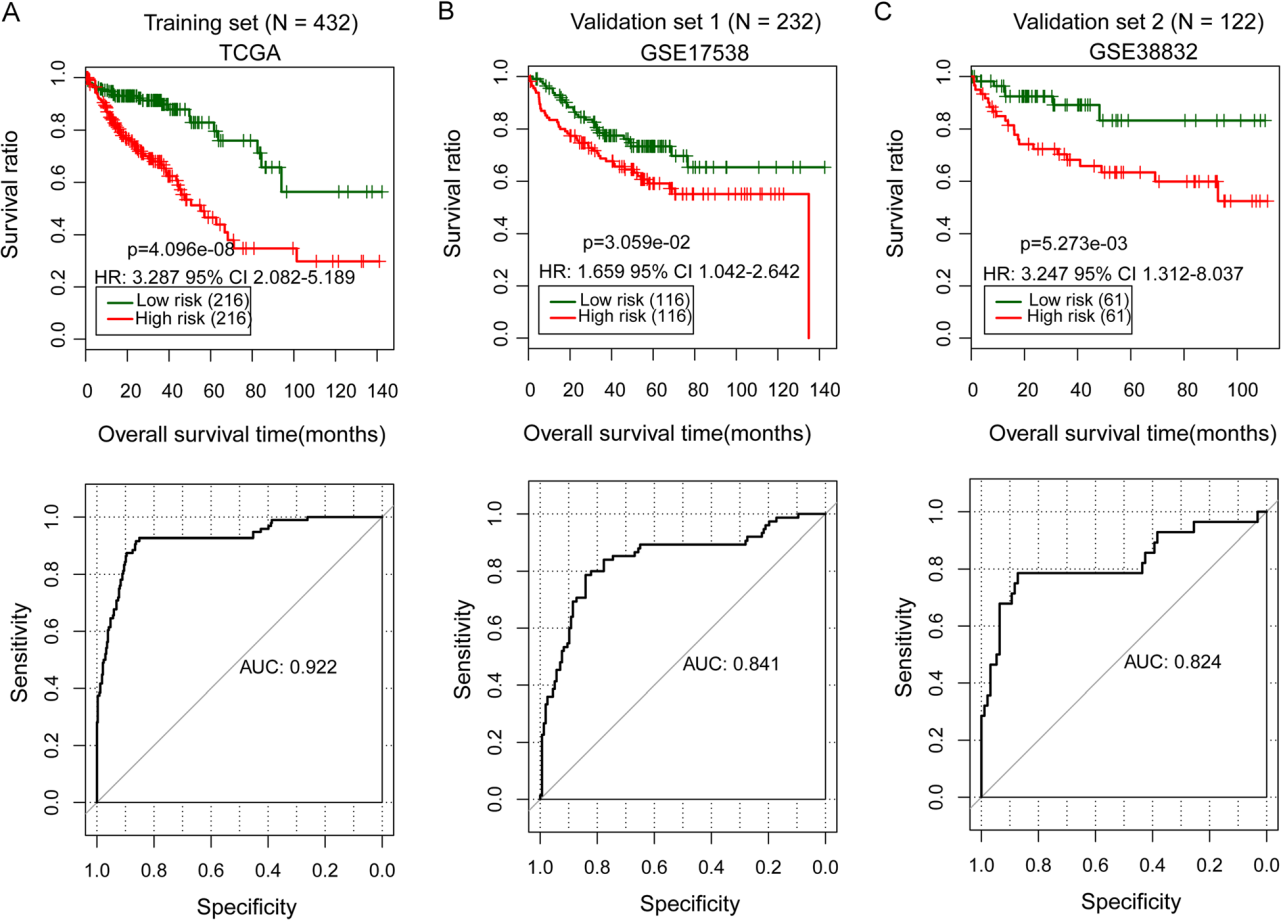
The Kaplan-Meier (K-M) survival analysis for samples with different risk scores. **a**–**c** The K-M survival analysis of samples in the low- and high- risk groups (upper), and the receiver operating characteristic (ROC) curve analysis for evaluating the prognostic model in predicting survival in the training (TCGA) and validation datasets(GSE44861 and GSE44076; lower). HR represents hazard ratio, and the number in parentheses indicates 95% confidence interval (CI). AUC, the area under the ROC curve

Based on the univariate Cox regression analysis, 84 prognosis-associated DEGs were identified in the TCGA training dataset. The multivariate Cox regression analysis showed that 14 out of the 84 DEGs were independently correlated with the prognosis of patients with colon cancer (Table S1). Afterward, an optimized prognostic gene signature was identified using the Cox-PH model, which consisted of 12 DEGs, including *ADORA3*, *CPA3*, *CPM*, *EDN3*, *FCRL2*, *MFNG*, *NAT1*, *PCSK5*, *PPARGC1A*, *PRRX2*, *TNFRSF17*, and *WDR78* (Table 2). Most of these 12 genes were in the blue (*n* = 5) and green modules (*n* = 6). The prognostic gene model of colon cancer was built according to the following algorithm: prognostic risk score = 0.44262 × Exp_*ADORA3*_ + (− 0.35894) × Exp_*CPA3*_ + (− 0.26349) × Exp_*CPM*_ + (− 0.12557) × Exp_*EDN3*_ + 1.38523 × Exp_*FCRL2*_ + 0.35734 × Exp_*MFNG*_ + (− 0.42755) × Exp_*NAT1*_ + 0.30206 × Exp_*PCSK5*_ + (− 0.34355) × Exp_*PPARGC1A*_ + 0.04376× Exp_*PRRX2*_ + (− 0.21594) × Exp_*TNFRSF17*_ + (− 0.07166) × Exp_*WDR78*_. The 432 samples in the TCGA training set were then divided into the high- (*n* = 216) and low-risk (*n* = 216) groups according to the median prognostic risk score. The K-M survival test indicated that patients with high-risk scores had a significantly shorter survival time compared with patients with low-risk scores (hazard ratio, HR = 3.287, 95% CI 2.082–5.189, *p* = 4.096e^−08^; Fig. 5a). The ROC curve analysis showed the prognostic model had a high accuracy in predicting the prognosis of colon cancer in the training set (area under the ROC curve, AUC = 0.922; Fig. 5a).

**Table 2:** The list of the differentially expressed genes in the optimized prognostic gene signature was identified by the Cox-proportional hazards (Cox-PH) model

Symbol	Module	Univariate Cox regression analysis			LASSO coefficient
		HR	95%CI	***P*** value	
ADORA3	Blue	1.570	1.067–2.549	3.40E−02	0.44262
CPA3	Blue	0.810	0.679–0.965	9.50E−03	− 0.35894
CPM	Green	0.748	0.561–0.995	2.30E−02	− 0.26349
EDN3	Green	0.830	0.670–1.028	4.40E−02	− 0.12557
FCRL2	Blue	2.465	1.298–4.682	2.90E−03	1.38523
MFNG	Blue	1.456	1.127–1.879	2.00E−03	0.35734
NAT1	Green	0.514	0.368–0.717	4.55E−05	− 0.42755
PCSK5	Green	1.477	1.021–2.138	1.95E−02	0.30206
PPARGC1A	Green	0.579	0.399–0.842	2.10E−03	− 0.34355
PRRX2	Yellow	1.260	1.017–1.559	1.70E−02	0.04376
TNFRSF17	Blue	0.780	0.597–0.919	3.45E−02	− 0.21594
WDR78	Green	0.334	0.158–0.707	2.05E−03	− 0.07166

*LASSO* L1-penalized least absolute shrinkage and selection operator, *HR* hazard ratio, *CI* confidential interval

### Validation of the prognostic model

Similarly, the samples with clinical overall survival data in the two validation datasets (GSE17538, *n* = 232; and GSE38832, *n* = 122) were separately divided into the high- and low-risk groups according to the prognostic risk scores (Fig. 5b, c). The K-M survival analysis showed there was a significant difference in the overall survival time between patients in the high and low groups in the two datasets (GSE17538: HR = 1.659, 95% CI 1.042–2.642, *p* = 3.059e^−02^; GSE38832: HR = 3.247, 95% CI 1.312–9.037, *p* = 5.273e^−03^; Fig. 5b, c). Besides, the model had high accuracies in predicting the prognosis in the two datasets (GSE17538: AUC = 0.841; GSE38832: AUC = 0.824). These results suggested the high performance of this model in predicting the prognosis of colon cancer.

### Identification of prognosis-associated clinical factors

**Fig. 6:**
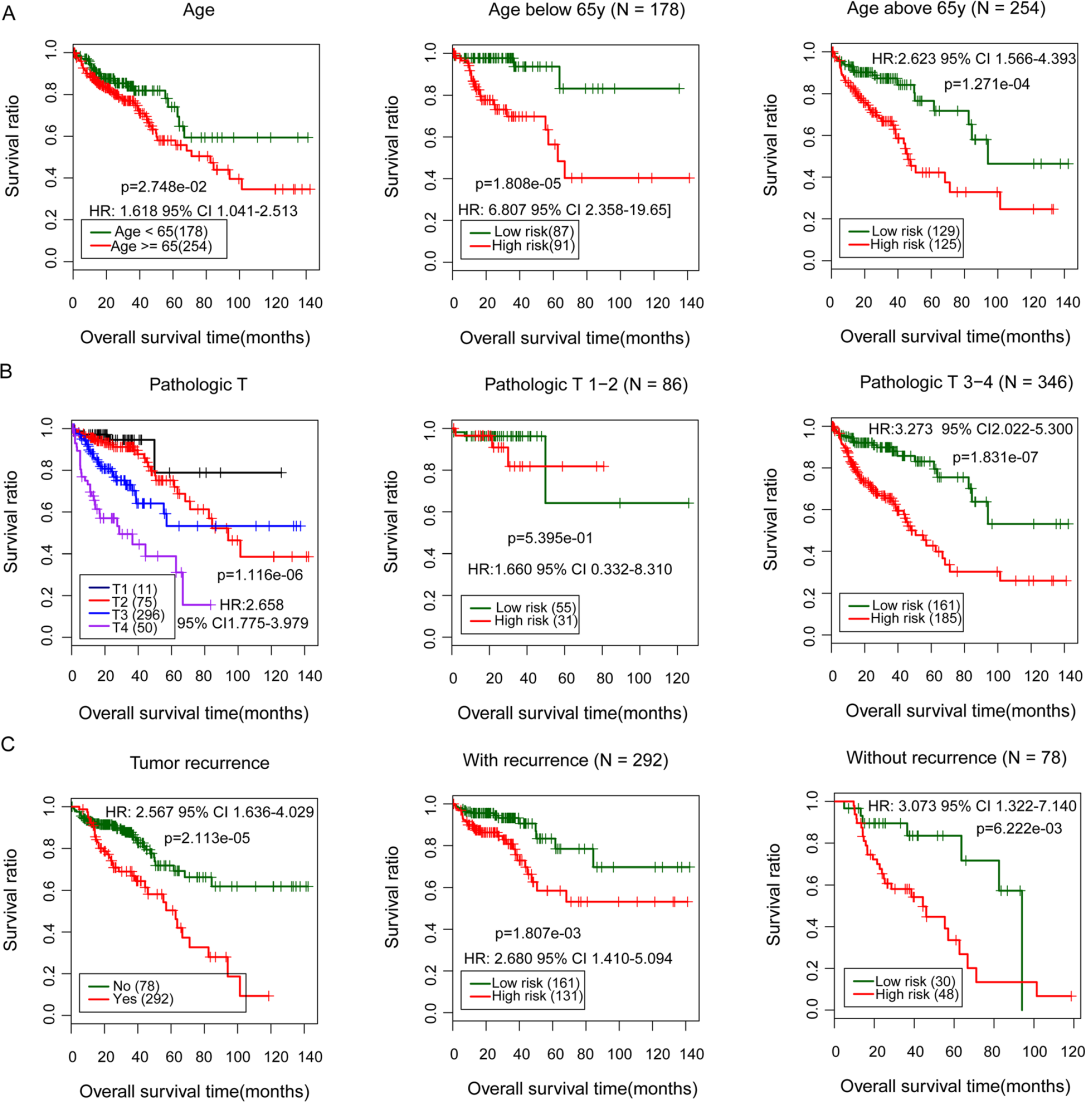
The subgroup Kaplan-Meier (K-M) survival analyses of prognosis-associated clinical factors analysis. **a**–**c** The K-M survival analysis of age, pathological T, and tumor recurrence in all samples (left), as well as different subgroups divided by the status of age, clinical T classification, and recurrence status (middle and right). HR represents hazard ratio, and the number in parentheses indicates 95% confidence interval (CI)

Before the construction of the nomogram model, the prognosis-associated clinical factors were identified using the univariate and multivariate Cox regression analysis. The stepwise Cox regression analyses showed that patient’s age (HR = 1.047, 95% CI 1.021–1.073, *p* = 3.510e^−04^), pathologic T classification (HR = 3.561, 95% CI 1.781–7.121, *p* = 3.280e^−04^), recurrence (HR = 1.881, 95% CI 1.050–3.369, *p* = 3.363e^−02^), and the risk model status (high/low; HR = 2.737, 95% CI 1.447–5.178, *p* = 1.970e^−03^) were prognosis-associated factors in the TCGA cohort (Table 3). The K-M survival analysis indicated that there was a significantly lower survival ratio in patients aged above 65 years (HR = 1.618, 95% CI 1.041–2.513, *p* = 2.748e^−02^; Fig. 6a, left), with advanced T classification (HR = 2.658, 95% CI 1.775-3.979, *p* = 1.116e^−06^; Fig. 6b, left), and with recurrence tumor (HR = 2.567, 95% CI 1.636–4.029, *p* = 2.113e^−05^; Fig. 6c, left) in comparison with the corresponding control groups, respectively. These results indicated the significant correlation of patients’ age, T classification, and recurrence status with the prognosis of colon cancer.

**Table 3:** Identification of the prognosis-associated factors in colon cancer (the TCGA samples) using Cox regression analysis

Clinical characteristics	TCGA (***N*** = 432)	Univariate		Multivariate	
		HR (95% CI)	***p*** value	HR (95% CI)	***p*** value
Age (years, mean ± sd)	66.78 ± 12.88	1.018 (1.001–1.035)	3.408E−02	1.047 (1.021–1.073)	3.510E−04
Gender (male/female)	230/202	1.077 (0.719–1.610)	7.189E−01	–	–
Pathologic M (M0/M1/–)	319/59/54	4.536 (2.851–7.218)	2.649E−12	1.501 (0.373–6.036)	5.671E− 01
Pathologic N (N0/N1/N2)	254/101/77	2.088 (1.648–2.644)	1.342E−10	1.614 (0.839–3.103)	1.514E−01
Pathologic T(T1/T2/T3/T4)	11/75/296/50	2.658 (1.775–3.979)	1.116E−06	3.561 (1.781–7.121)	3.280E−04
Pathologic stage (I/II/III/IV/–)	73/167/123/59/10	2.181 (1.719–2.767)	3.376E−11	1.123 (0.373–3.378)	8.362E−01
Colon polyps history (yes/no/–)	128/239/65	0.731 (0.426–1.255)	2.537E− 01	–	–
Lymphatic invasion (yes/no/–)	150/241/41	2.150 (1.392–3.320)	4.125E− 04	0.922 (0.489–1.737)	8.024E− 01
Recurrence (yes/no)	78/292/62	2.567 (1.636–4.029)	2.113E−05	1.881 (1.050–3.369)	3.363E− 02
PS model status (high/low)	216/216	3.287 (2.082–5.189)	4.096E−08	2.737 (1.447–5.178)	1.970E−03
Vital status (dead/alive)	96/336	–	–	–	–
Overall survival time (months, mean ± sd)	29.44 ± 25.43	–	–	–	–

*HR* hazard ratio, *CI* confidential interval, *TCGA* The Cancer Genome Atlas, *SD* standard deviation

Besides, the subgroup K-M survival analysis showed that high risk score was correlated with a lower survival ratio in patients aged below 65 years (HR = 6.807, 95% CI 2.358–19.65, *p* = 1.808e^−05^; Fig. 6a, middle), aged above 65 years (HR = 2.623, 95% CI 1.566–4.393, *p* = 1.271e^−04^; Fig. 6a, right), with advanced T classifications (T13-4, HR = 3.273, 95% CI 2.022–5.300, *p* = 1.831e^−07^; Fig. 6b, right), with tumor recurrence (HR = 2.680, 95% CI 1.410–5.094; *p* = 1.807e^−03^; Fig. 6c, middle), and without tumor recurrence (HR = 3.073, 95% CI 1.322–7.140; *p* = 6.222e^−03^; Fig. 6c, right). For patients with early T classifications (T1-2), there was no difference in the survival ratio between patients with high- and low-risk scores (HR = 1.660, *p* = 5.395e^−01^; Fig. 6b, middle). The subgroup analysis indicated that the prognostic gene model had high performance in predicting the prognosis of patients with colon cancer, irrespective of the clinical age and tumor recurrence status.

### Nomogram model construction

**Fig. 7:**
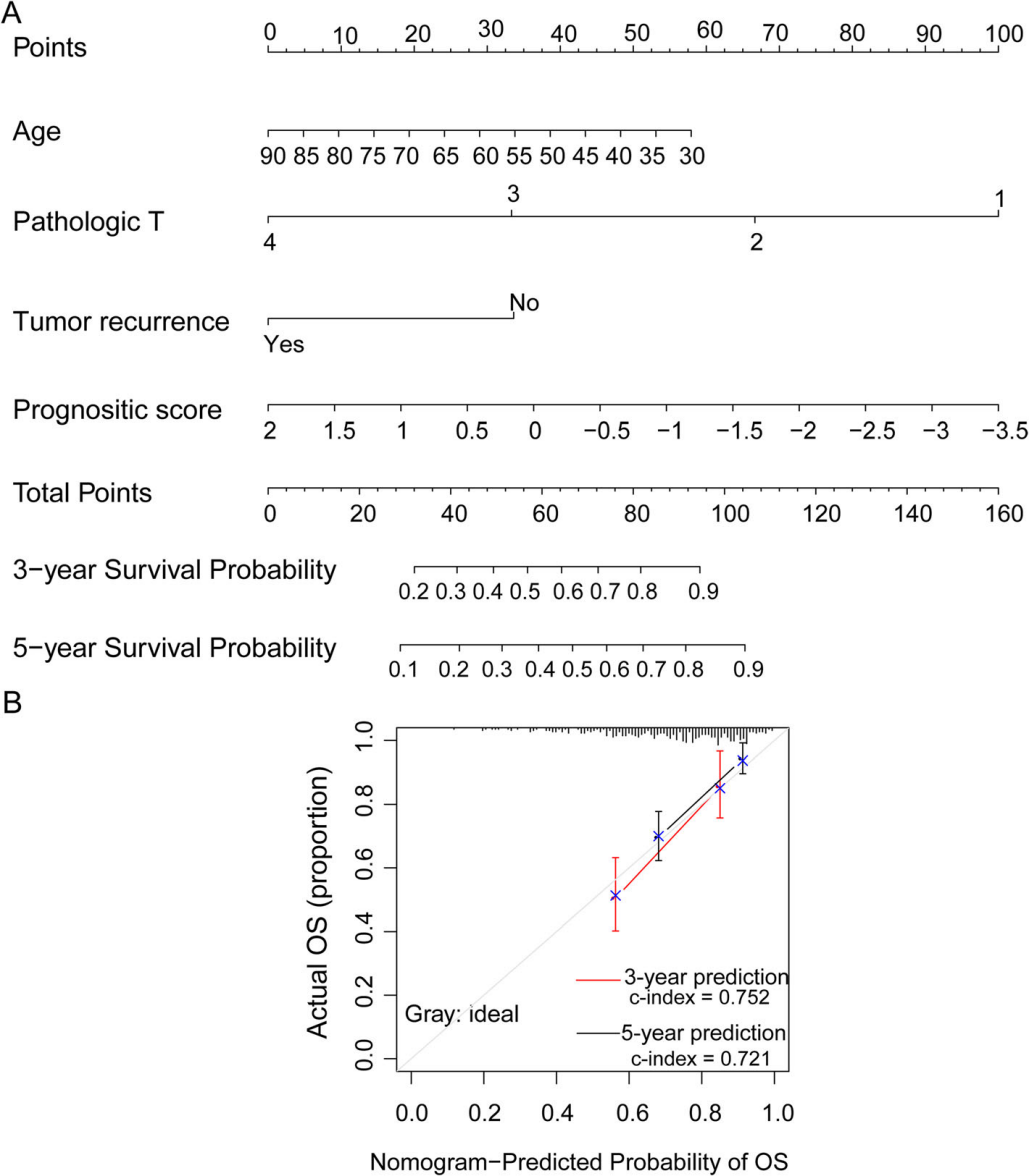
The nomogram model analysis. **a** The predictive weight of each factor and prognostic risk score in predicting the prognosis of colon cancer. The red line with arrow notes the 3- and 5-year survival probability of the example case. **b** The difference analysis between nomogram-predicted survival probability and the actual survival. The nomogram-predicted survival probabilities have high compliances with the actual situations (c-index = 0.752 and 0.721)

According to the above analyses, the nomogram model was constructed using the prognosis-associated factors, including patients’ age, clinical T classification, and tumor recurrence status (Fig. 7a). According to the nomogram, we found that patients with older age, an advanced T classification, tumor recurrence, and a high risk score had low 3- and 5-year survival probabilities. Take an 85-year-old man (~ 5 points), with T3 classification (~ 33.7 points), with tumor recurrence (0 points), and a risk score of 1.5 (~ 9.3 points), for example, he had a total point of 48. His 3- and 5-year survival probabilities were approximately 40% and 28%, respectively (Fig. 7a). What’s more, the predicted 3- and 5-year survival probabilities had high compliance with the actual situations (c-index = 0.752 and 0.721; Fig. 7b). These results suggested the clinical applicability of this prognostic model in predicting the prognosis of colon cancer.

### The features of the DEGs between patients with different prognosis risk scores

**Fig. 8:**
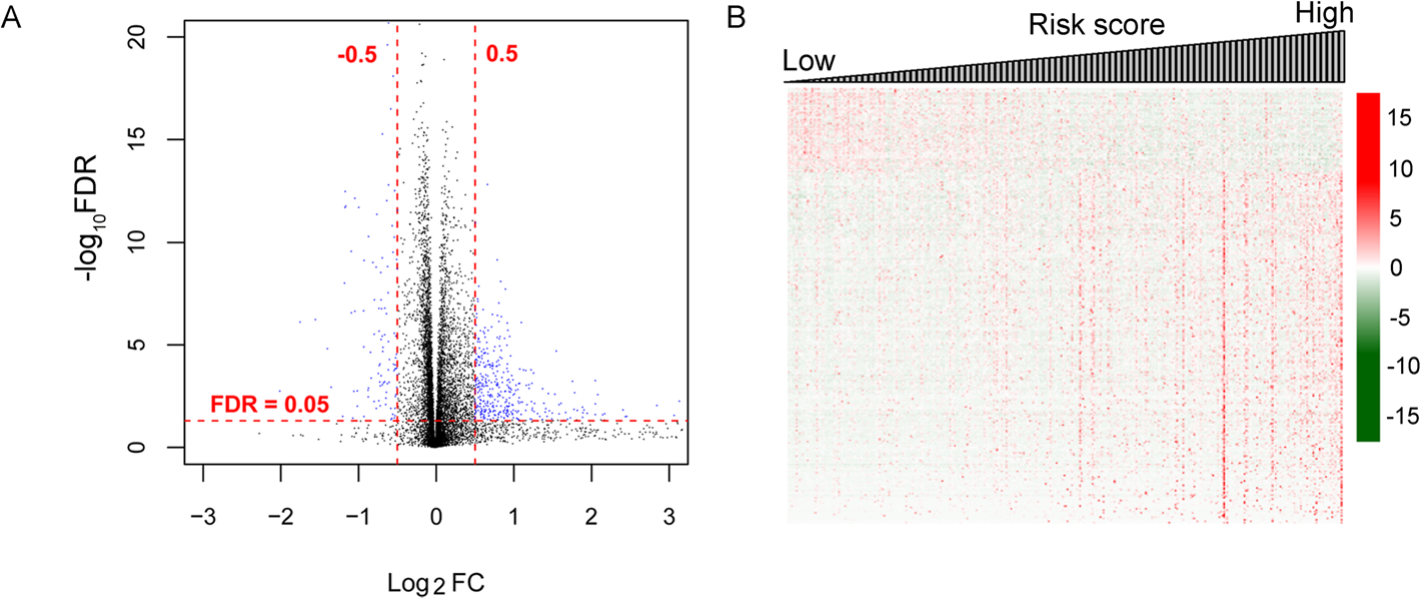
Screening of differentially expressed genes (DEGs) in the TCGA samples with high- and low-prognostic risk scores. **a** The scatter plot of the 514 DEGs between the high- and low-risk groups. Blue nodes indicate genes are upregulated (FDR < 0.05 and log_2_FC > 0.5) and downregulated DEGs (FDR < 0.05 and log_2_FC < − 0.5). **b** The sample heatmap of the 514 DEGs in the TCGA cohort (*n* = 432). FDR, false discovery rate. FC, fold change. TCGA, The Cancer Genome Atlas

At last, we investigated the differential gene expression profiles between TCGA samples with high- and low-risk scores. A total of 514 DEGs were identified between high- and low-risk groups, including 102 downregulated and 412 upregulated genes (Fig. 8a). The clustering analysis indicated that the expression profiles of these DEGs changed with the risk scores (Fig. 8b), showing the co-expression profiles of these DEGs with the 12-gene signature.

## Discussion

In the present study, 5 significantly stable gene modules (including 1160 genes) related to colon cancer were constructed by the WGCNA algorithm. Then, 1153 common DEGs across the TCGA, GSE44861, and GSE44076 datasets were identified between colon cancer tumor and normal tissue samples. Furthermore, the expression features of 12 prognosis-associated DEGs (*ADORA3*, *CPA3*, *CPM*, *EDN3*, *FCRL2*, *MFNG*, *NAT1*, *PCSK5*, *PPARGC1A*, *PRRX2*, *TNFRSF17*, and *WDR78*) were identified as the optimized prognostic gene signature. The corresponding prognostic model presented high performance for predicting the prognosis of colon cancer both in the training dataset and in the validation datasets. Besides, we found that the predicted 3- and 5-year survival probabilities using the combination of the model status with clinical factors (including patients’ age, pathological T classification, and tumor recurrence status) showed high compliance with the actual 3- and 5-year overall survival proportion. These results indicated that the prognostic gene signature was of great reference value for predicting the prognosis and survival probability of colon cancer.

The advances in mining the genetic properties of various diseases have been enhanced due to the rapid technological development in high-throughput sequencing and bioinformatics [34]. The GEO and TCGA databases, as public available cancer genomic databases, provide the comprehensive data of cancers, including mRNA expression data, miRNA expression data, copy number variation, DNA methylation, and clinical information [35, 36]. The TCGA and GEO data have been effectively applied to improve diagnostic and therapeutic methods and potential of cancers [35–37]. Thus, this study was performed based on the gene expression profile data and clinical information of colon cancer retrieved from the TCGA and GEO databases. Gene expression profiles have been reported to predict the prognosis outcome of cancers [38–40]. Computationally, the Cox regression methods were commonly used to construct the prognostic models and screen prognostic factors [41]. The availability of this model in survival analysis has been confirmed in recent studies [42, 43]. Similarly, in this study, the Cox regression model based on the LASSO was applied to screen the optimized gene set with potential prognostic value. The 12-gene prognostic signature constructed by the LASSO Cox regression model showed a higher predictive ability both in the TCGA training data and the two validation sets (GSE17538 and GSE38832; AUC > 0.800).

Besides, this study showed that age, pathological T classification, and tumor recurrence were prognosis-associated factors in patients with colon cancer. Consistent with our results, previous studies have also demonstrated that older age, advanced pathological T, and tumor recurrence are associated with poor prognosis in patients with colon cancer [44–46]. Notably, the nomogram analysis in the current study revealed that the combination of patients’ age, T classification, recurrence status, and prognostic risk score had 3- and 5-year survival probabilities close to actual clinical situations. These results further showed that the 12-gene prognostic model had a significant predictive ability for the prognosis of colon cancer.

In this study, the prognostic model was constructed based on the signature of 12 prognosis-associated genes, including 12 DEGs, *ADORA3*, *CPA3*, *CPM*, *EDN3*, *FCRL2*, *MFNG*, *NAT1*, *PCSK5*, *PPARGC1A*, *PRRX2*, *TNFRSF17*, and *WDR78*. Specifically, the adenosine receptor A3 (ADORA3) protein encoded by the *ADORA3* gene is a G-protein-coupled receptor that functions in inflammatory and immunological responses as well as cancer growth through influencing the nucleotide metabolic process [47–49]. There is increasing evidence proving that ADORA3 is overexpressed in several cancers, including breast cancer [50], thyroid cancer [51], bladder cancer [52], and colon cancer [53] and functions as a tumor promoter [54]. Carboxypeptidase A3 (CPA3) is a member of the CPA family of zinc metalloproteases released by mast cells and may be involved in the inactivation of venom-associated peptides and the degradation of endogenous proteins [55]. Previous studies have shown the elevated expression of CPA3 in asthma [56] and anaphylactic shock [57]. However, few studies have investigated the role of CPA3 in cancers. CPM is also an arginine/lysine CP which exerts important roles in angiogenesis, proliferation, and apoptosis through modulating chemokines or kinins in cancer cells [58]. Notably, a recent study reports that CPM/Src-FAK pathway is involved in cell migration and invasion in colon cancer [59]. Endothelin 3 (END3) is reported to participate in the progression of several cancers including malignant melanoma [60], cervical cancer [61], and colon cancer [62]. Fc Receptor Like 2 (FCRL2) is a member of the immunoglobulin receptor superfamily that is involved in the development of lymphoblastic leukemia by immunomodulating B cell function [63–65]. Besides, it has been reported that the inherited polymorphism in the acetyltransferase 1 (NAT1) gene increases the risk of colorectal adenocarcinoma [66]. Manic fringe (MFNG) is reported to exhibit antitumor effects in lung cancer [67]. The peroxisome proliferator-activated receptor-γ coactivator 1-α (PPARGC1A) gene also contributes to tumor growth and metastasis in several cancers [68, 69]. In addition, studies have suggested that both the paired related homeobox 2 (PRRX2) gene [70, 71] and the tumor necrosis factor receptor superfamily member 17 (TNFRSF17) gene [72, 73] are associated with the development of several cancers, while the proprotein convertase subtilisin/kexin type 5 (PCSK5) gene and the WD repeat domain 78 (WDR78) gene have not been reported to be associated with pathogenesis and progression. Thus, the functions of these genes in colon cancer should be further investigated using preclinical and clinical experiments.

## Conclusions

In conclusion, the prognostic model based on the signature of the 12 genes (*ADORA3*, *CPA3*, *CPM*, *EDN3*, *FCRL2*, *MFNG*, *NAT1*, *PCSK5*, *PPARGC1A*, *PRRX2*, *TNFRSF17*, and *WDR78*) exhibited a relatively satisfactory and credible predictive power for the prognosis of colon cancer, making it a great potential biomarker. However, the prognostic significance and practicability of the 12-gene prognostic model in colon cancer should be further confirmed in clinical studies.

## Supplementary Information

**Additional file 1: Figure S1.** Weighed gene co-expression network analysis in the training (TCGA) and validation (GSE44861 and GSE44076) datasets. (A) The correlation between the gene expression profiles in the TCGA, GSE44861 and GSE44076 datasets. Upper: the correlation analysis of gene expression level in the training and validation datasets; Lower: the analysis of node connection in the training and validation datasets. (B) Scale independence of the weighted gene co-expression network analysis (WGCNA). Left: the diagram for selecting the soft threshold power: The x axis represents the power of the weighting parameter and the y axis represents the scale-free topology model fit signed R^2^ in the network; Right: The diagram of gene connectivity corresponds to power value. The red line indicates the value under different power parameter and the average node connectivity of 1.

**Additional file 2: Table S1.** The list of the prognosis-associated differentially expressed genes across the three datasets (TCGA, GSE44861, and GSE44076) using the Cox regression analysis.

## Data Availability

GSE17538, GSE38832, GSE44861, and GSE44076 datasets were preliminarily extracted from the National Center for Biotechnology Information (NCBI) gene expression omnibus (GEO) repository (https://www.ncbi.nlm.nih.gov/geo/). Additional transcriptome RNA expression data of colon cancer were downloaded from TCGA (https://gdc-portal.nci.nih.gov/). All data generated or analyzed during this study are included in this published article.

## Abbreviations

Cox-PH: Cox-proportional hazards
DEGs: Differentially expressed genes
FC: Fold change
FDR: False discovery rate
GEO: Gene expression omnibus
KEGG: Kyoto Encyclopedia of Genes and Genomes
NCBI: National Center for Biotechnology Information
TCGA: The Cancer Genome Atlas
WGCNA: Weighted gene co-expression network analysis

## References

[1] Siegel RL, Miller KD, Jemal A (2019). Cancer statistics, 2019. CA: Cancer J Clinicians.

[2] Bray F, Ferlay J, Soerjomataram I, Siegel RL, Torre LA, Jemal A (2018). Global cancer statistics 2018: GLOBOCAN estimates of incidence and mortality worldwide for 36 cancers in 185 countries. CA: Cancer J Clinicians.

[3] Hashiguchi Y, Muro K, Saito Y, Ito Y, Ajioka Y, Hamaguchi T, Hasegawa K, Hotta K, Ishida H, Ishiguro M. Japanese Society for Cancer of the Colon and Rectum (JSCCR) guidelines 2019 for the treatment of colorectal cancer. Int J Clin Oncol 2020;2019:1-42. 10.1007/s10147-019-01485-z PMC6946738 31203527

[4] Bagante F, Spolverato G, Beal E, Merath K, Chen Q, Akgül O, Anders RA, Pawlik TM (2018). Impact of histological subtype on the prognosis of patients undergoing surgery for colon cancer. J Surg Oncol.

[5] Fujikawa H, Toiyama Y, Inoue Y, Imaoka H, Shimura T, Okigami M, Yasuda H, Hiro J, Yoshiyama S, Saigusa S (2017). Prognostic impact of preoperative albumin–to–globulin ratio in patients with colon cancer undergoing surgery with curative intent. Anticancer Research.

[6] Nagata H, Ishihara S, Hata K, Murono K, Kaneko M, Yasuda K, Otani K, Nishikawa T, Tanaka T, Kiyomatsu T (2017). Survival and prognostic factors for metachronous peritoneal metastasis in patients with colon cancer. Ann Surgical Oncol.

[7] Wang Y, He S, Zhu X, Qiao W, Zhang J (2016). High copy number of mitochondrial DNA predicts poor prognosis in patients with advanced stage colon cancer. Int J Biological Markers.

[8] Yokota M, Kojima M, Higuchi Y, Nishizawa Y, Kobayashi A, Ito M, Saito N, Ochiai A (2016). Gene expression profile in the activation of subperitoneal fibroblasts reflects prognosis of patients with colon cancer. Int J Cancer.

[9] Sanoff HK, Sargent DJ, Campbell ME, Morton RF, Fuchs CS, Ramanathan RK, Williamson SK, Findlay BP, Pitot HC, Goldberg RM (2008). Five-year data and prognostic factor analysis of oxaliplatin and irinotecan combinations for advanced colorectal cancer: N9741. J Clin Oncol.

[10] Sun Q, Liu P, Long B, Zhu Y, Liu T. Screening of significant biomarkers with poor prognosis in hepatocellular carcinoma via bioinformatics analysis. Medicine. 2020;99:e21702. 10.1097/MD.0000000000021702 PMC7593045 32769939

[11] Fakih M, Ouyang C, Wang C, Tu TY, Gozo MC, Cho M, Sy M, Longmate JA, Lee PP (2019). Immune overdrive signature in colorectal tumor subset predicts poor clinical outcome. J Clin Investigation.

[12] Tu M, Wang X, Chen P, Li J, Luo X, He L, Huang W, Hong J, Qu C (2020). RCE1 deficiency enhances invasion via the promotion of epithelial-mesenchymal transition and predicts poor prognosis in hepatocellular carcinoma. Am J Transl Res.

[13] Hansen TF, Kjær-Frifeldt S, Eriksen AC, Lindebjerg J, Jensen LH, Sørensen FB, Jakobsen A (2018). Prognostic impact of CDX2 in stage II colon cancer: results from two nationwide cohorts. Brit J Cancer.

[14] Zhang Q-N, Zhu H-L, Xia M-T, Liao J, Huang X-T, Xiao J-W, Yuan C (2019). A panel of collagen genes are associated with prognosis of patients with gastric cancer and regulated by microRNA-29c-3p: An integrated bioinformatics analysis and experimental validation. Cancer Management Research.

[15] Dalerba P, Sahoo D, Paik S, Guo X, Yothers G, Song N, Wilcox-Fogel N, Forgó E, Rajendran PS, Miranda SP (2016). CDX2 as a prognostic biomarker in stage II and stage III colon cancer. New England J Med.

[16] Wang X, Tan C, Ye M, Wang X, Weng W, Zhang M, Ni S, Wang L, Huang D, Huang Z (2020). Development and validation of a DNA repair gene signature for prognosis prediction in Colon Cancer. J Cancer.

[17] Chen J, He Q, Wu P, Fu J, Xiao Y, Chen K, Xie D, Zhang X. ZMYND8 expression combined with pN and pM classification as a novel prognostic prediction model for colorectal cancer: based on TCGA and GEO database analysis. Cancer Biomarkers. 2020;28:201–11. 10.3233/CBM-191261 PMC12662352 32224527

[18] Zou J, Duan D, Yu C, Pan J, Xia J, Yang Z, Cai S (2020). Mining the potential prognostic value of synaptosomal-associated protein 25 (SNAP25) in colon cancer based on stromal-immune score. PeerJ.

[19] Zheng W, Yang C, Qiu L, Feng X, Sun K, Deng H. Transcriptional information underlying the generation of CSCs and the construction of a nine-mRNA signature to improve prognosis prediction in colorectal cancer. Cancer Biology Therapy. 2020;20:688–97. 10.1080/15384047.2020.1762419 PMC7515529 32453965

[20] Zhang R, Ye J, Huang H, Du X (2019). Mining featured biomarkers associated with vascular invasion in HCC by bioinformatics analysis with TCGA RNA sequencing data. Biomed Pharmacotherapy.

[21] Al-Sheikh YA, Ghneim HK, Alharbi KK, Aboul-Soud MA (2019). Screening for differentially-expressed microRNA biomarkers in Saudi colorectal cancer patients by small RNA deep sequencing. Int J Molecular Medicine.

[22] Yamada A, Yu P, Lin W, Okugawa Y, Boland CR, Goel A (2018). A RNA-Sequencing approach for the identification of novel long non-coding RNA biomarkers in colorectal cancer. Scientific Reports.

[23] Besso MJ, Montivero L, Lacunza E, Argibay MC, Abba M, Furlong LI, Colas E, Gil-Moreno A, Reventos J, Bello R (2020). Identification of early stage recurrence endometrial cancer biomarkers using bioinformatics tools. Oncology Reports.

[24] Zhai X, Xue Q, Liu Q, Guo Y, Chen Z (2017). Colon cancer recurrence-associated genes revealed by WGCNA co-expression network analysis. Mole Med Reports.

[25] Langfelder P, Horvath S (2008). WGCNA: an R package for weighted correlation network analysis. BMC Bioinformatics.

[26] Qi C, Hong L, Cheng Z, Yin Q (2016). Identification of metastasis-associated genes in colorectal cancer using metaDE and survival analysis. Oncology letters.

[27] Wang SB, Tan Y, Lei W, Wang YG, Zhou XM, Jia XY, Zhang KJ, Chu L, Liu XY, Qian WB (2012). Complete eradication of xenograft hepatoma by oncolytic adenovirus ZD55 harboring TRAIL-IETD-Smac gene with broad antitumor effect. Human Gene Therapy.

[28] Sherman BT, Lempicki RA (2009). Systematic and integrative analysis of large gene lists using DAVID bioinformatics resources. Nature Protocols.

[29] Huang DW, Sherman BT, Lempicki RA (2008). Bioinformatics enrichment tools: paths toward the comprehensive functional analysis of large gene lists. Nucleic Acids Research.

[30] Wang P, Wang Y, Hang B, Zou X, Mao J-H (2016). A novel gene expression-based prognostic scoring system to predict survival in gastric cancer. Oncotarget.

[31] Tibshirani R (1997). The lasso method for variable selection in the Cox model. Statistics In Medicine.

[32] Goeman JJ (2010). L1 penalized estimation in the Cox proportional hazards model. Biometrical Journal.

[33] Ritchie ME, Phipson B, Wu D, Hu Y, Law CW, Shi W (2015). Smyth GK: limma powers differential expression analyses for RNA-sequencing and microarray studies. Nucleic Acids Research.

[34] Vamathevan J, Birney E: A review of recent advances in translational bioinformatics: bridges from biology to medicine. Yearb Med Inform. 2017;26:178–87. 10.15265/IY-2017-017 PMC6239226 29063562

[35] Hutter C, Zenklusen JC (2018). The cancer genome atlas: creating lasting value beyond its data. Cell.

[36] Jiang P, Liu XS (2015). Big data mining yields novel insights on cancer. Nature Genetics.

[37] Liu X, Wang J, Chen M, Liu S, Yu X, Wen F: Combining data from TCGA, GEO database, and RT-qPCR validation to identify gene prognostic marker in lung cancer. In C74 lung cancer: biomarkers for prognosis and outcomes. Am Thoracic Society; 2019: A5549.

[38] Kessous R, Octeau D, Klein K, Tonin PN, Greenwood CM, Pelmus M, Laskov I, Kogan L, Salvador S, Lau S (2018). Distinct homologous recombination gene expression profiles after neoadjuvant chemotherapy associated with clinical outcome in patients with ovarian cancer. Gynecologic Oncol.

[39] O’Mara TA, Zhao M, Spurdle AB (2016). Meta-analysis of gene expression studies in endometrial cancer identifies gene expression profiles associated with aggressive disease and patient outcome. Scientific Reports.

[40] McConkey DJ, Choi W, Shen Y, Lee I-L, Porten S, Matin SF, Kamat AM, Corn P, Millikan RE, Dinney C (2016). A prognostic gene expression signature in the molecular classification of chemotherapy-naive urothelial cancer is predictive of clinical outcomes from neoadjuvant chemotherapy: a phase 2 trial of dose-dense methotrexate, vinblastine, doxorubicin, and cisplatin with bevacizumab in urothelial cancer. European Urology.

[41] Bao Z, Zhang W, Dong D (2017). A potential prognostic lncRNA signature for predicting survival in patients with bladder urothelial carcinoma. Oncotarget.

[42] Ching T, Zhu X, Garmire LX (2018). Cox-nnet: an artificial neural network method for prognosis prediction of high-throughput omics data. Plos Computational Biology.

[43] Liang R, Wang M, Zheng G, Zhu H, Zhi Y, Sun Z (2018). A comprehensive analysis of prognosis prediction models based on pathway-level, gene-level and clinical information for glioblastoma. International Journal Of Molecular Medicine.

[44] Di Fabio F, Nascimbeni R, Villanacci V, Baronchelli C, Bianchi D, Fabbretti G, Casella C, Salerni B (2004). Prognostic variables for cancer-related survival in node-negative colorectal carcinomas. Digestive Surgery.

[45] De Leon MP, Sant M, Micheli A, Sacchetti C, Gregorio CD, Fante R, Zanghieri G, Melotti G, Gatta G (1992). Clinical and pathologic prognostic indicators in colorectal cancer. A population-based study. Cancer.

[46] Roth AD, Delorenzi M, Tejpar S, Yan P, Klingbiel D, Fiocca R, d’Ario G, Cisar L, Labianca R, Cunningham D (2012). Integrated analysis of molecular and clinical prognostic factors in stage II/III colon cancer. J National Cancer Institute.

[47] Jacobson KA, Merighi S, Varani K, Borea PA, Baraldi S, Aghazadeh Tabrizi M, Romagnoli R, Baraldi PG, Ciancetta A, Tosh DK (2018). A3 adenosine receptors as modulators of inflammation: from medicinal chemistry to therapy. Med Res Reviews.

[48] Cohen S, Fishman P (2019). Targeting the A3 adenosine receptor to treat cytokine release syndrome in cancer immunotherapy. Drug Design Development Therapy.

[49] Gessi S, Merighi S, Borea PA, Cohen S, Fishman P. Adenosine Receptors and Current Opportunities to Treat Cancer. In: Borea P, Varani K, Gessi S, Merighi S, Vincenzi F (eds). The Adenosine Receptors. The Receptors, vol 34. Cham: Humana Press; 2018 10.1007/978-3-319-90808-3_23.

[50] Jafari SM, Panjehpour M, Aghaei M, Joshaghani HR, Enderami SE (2017). A3 adenosine receptor agonist inhibited survival of breast cancer stem cells via GLI-1 and ERK1/2 pathway. Journal Of Cellular Biochemistry.

[51] Morello S, Petrella A, Festa M, Popolo A, Monaco M, Vuttariello E, Chiappetta G, Parente L, Pinto A (2008). Cl-IB-MECA inhibits human thyroid cancer cell proliferation independently of A3 adenosine receptor activation. Cancer Biol Therapy.

[52] Cao H-L, Liu Z-J, Chang Z (2017). Cordycepin induces apoptosis in human bladder cancer cells via activation of A3 adenosine receptors. Tumor Biol.

[53] Gessi S, Cattabriga E, Avitabile A, Lanza G, Cavazzini L, Bianchi N, Gambari R, Feo C, Liboni A, Gullini S (2004). Elevated expression of A3 adenosine receptors in human colorectal cancer is reflected in peripheral blood cells. Clin Cancer Research.

[54] Marucci G, Santinelli C, Buccioni M, Navia AM, Lambertucci C, Zhurina A, Yli-Harja O, Volpini R, Kandhavelu M (2018). Anticancer activity study of A3 adenosine receptor agonists. Life Sciences.

[55] Springman EB: Mast cell carboxypeptidase. In Handbook of Proteolytic Enzymes. London: Academic Press. 2004:828-83.

[56] Abadalkareem R, Lau LC, Abdelmotelb A, Zhou X, Eren E, Walls AF (2017). Mast cell tryptase and carboxypeptidase A3 (CPA3) as markers for predicting susceptibility to severe allergic drug reactions. J Allergy Clin Immunol.

[57] Yang K, Guo X, Yan X, Gao C (2012). Changes of prostaglandin D2, carboxypeptidase A3 and platelet activating factor in guinea pig in anaphylactic shock. Fa Yi Xue Za Zhi.

[58] Denis CJ, Lambeir A-M (2013). The potential of carboxypeptidase M as a therapeutic target in cancer. Expert Opinion Therapeutic Targets.

[59] Lu D, Yao Q, Zhan C, Le-Meng Z, Liu H, Cai Y, Tu C, Li X, Zou Y, Zhang S (2017). MicroRNA-146a promote cell migration and invasion in human colorectal cancer via carboxypeptidase M/src-FAK pathway. Oncotarget.

[60] Tang L, Su M, Zhang Y, Ip W, Martinka M, Huang C, Zhou Y (2008). Endothelin-3 is produced by metastatic melanoma cells and promotes melanoma cell survival. J Cutaneous Med Surg.

[61] Sun DJ, Liu Y, Lu DC, Kim W, Lee JH, Maynard J, Deisseroth A (2007). Endothelin-3 growth factor levels decreased in cervical cancer compared with normal cervical epithelial cells. Human Pathology.

[62] Olender J, Nowakowska-Zajdel E, Kruszniewska-Rajs C, Orchel J, Mazurek U, Wierzgoń A, Kokot T, Muc-Wierzgoń M (2016). Epigenetic silencing of endothelin-3 in colorectal cancer. Int J Immunopathol Pharmacol.

[63] Ehrhardt GR, Leu C-M, Zhang S, Aksu G, Jackson T, Haga C, Hsu JT, Schreeder DM, Davis RS, Cooper MD: Fc receptor–like proteins (FCRL): immunomodulators of B cell function. In Mechanisms of Lymphocyte Activation and Immune Regulation XI. Boston: Springer; 2007. p. 155-16. 10.1007/0-387-46530-8_14 17338184

[64] Kazemi T, Asgarian-Omran H, Memarian A, Shabani M, Sharifian RA, Vossough P, Ansaripour B, Rabbani H, Shokri F (2009). Low representation of Fc receptor-like 1–5 molecules in leukemic cells from Iranian patients with acute lymphoblastic leukemia. Cancer Immunol Immunotherapy.

[65] Kazemi T, Asgarian-Omran H, Hojjat-Farsangi M, Shabani M, Memarian A, Sharifian RA, Razavi SM, Jeddi-Tehrani M, Rabbani H, Shokri F (2008). Fc receptor-like 1–5 molecules are similarly expressed in progressive and indolent clinical subtypes of B-cell chronic lymphocytic leukemia. Int J Cancer.

[66] Katoh T, Boissy R, Nagata N, Kitagawa K, Kuroda Y, Itoh H, Kawamoto T, Bell DA (2000). Inherited polymorphism in the N-acetyltransferase 1 (NAT1) and 2 (NAT2) genes and susceptibility to gastric and colorectal adenocarcinoma. Int J Cancer.

[67] Yi F, Amarasinghe B, Dang TP (2013). Manic fringe inhibits tumor growth by suppressing Notch3 degradation in lung cancer. Am J Cancer Research.

[68] Andrzejewski S, Klimcakova E, Johnson RM, Tabariès S, Annis MG, McGuirk S, Northey JJ, Chénard V, Sriram U, Papadopoli DJ (2017). PGC-1α promotes breast cancer metastasis and confers bioenergetic flexibility against metabolic drugs. Cell Metabolism.

[69] Li Y, Xu S, Li J, Zheng L, Feng M, Wang X, Han K, Pi H, Li M, Huang X (2016). SIRT1 facilitates hepatocellular carcinoma metastasis by promoting PGC-1α-mediated mitochondrial biogenesis. Oncotarget.

[70] Juang YL, Jeng YM, Chen CL, Lien HC (2016). PRRX2 as a novel TGF-β-induced factor enhances invasion and migration in mammary epithelial cell and correlates with poor prognosis in breast cancer. Molecular Carcinogenesis.

[71] Wang Q, Chen D-L, Zhang L-F, Bian H (2018). Promoting cell viability and migration of gastric cancer cells by PRRX2 via activation of Wnt/β-catenin signaling pathway. Chinese J Pathophysiol.

[72] Castanas E, Kampa M, Pelekanou V, Notas G, Athanasouli P, Alexakis K, Kagiadaki F, Peroulis N, Kalyvianaki K, Kampouri E (2018). BCMA (TNFRSF17) induces APRIL and BAFF mediated breast cancer cell stemness. Front Oncol.

[73] Chae S-C, Yu J-I, Uhm T-B, Lee S-Y, Kang D-B, Lee J-K, Park W-C, Yun K-J (2012). The haplotypes of TNFRSF17 polymorphisms are associated with colon cancer in a Korean population. Int J Colorectal Disease.

