# Supplementary figures and images for: Identification of a prognostic gene signature of colon cancer using integrated bioinformatics analysis
Source: World J Surg Oncol. 2021 Jan 13;19:13. doi: 10.1186/s12957-020-02116-y (PMC7807455; doi:10.1186/s12957-020-02116-y)

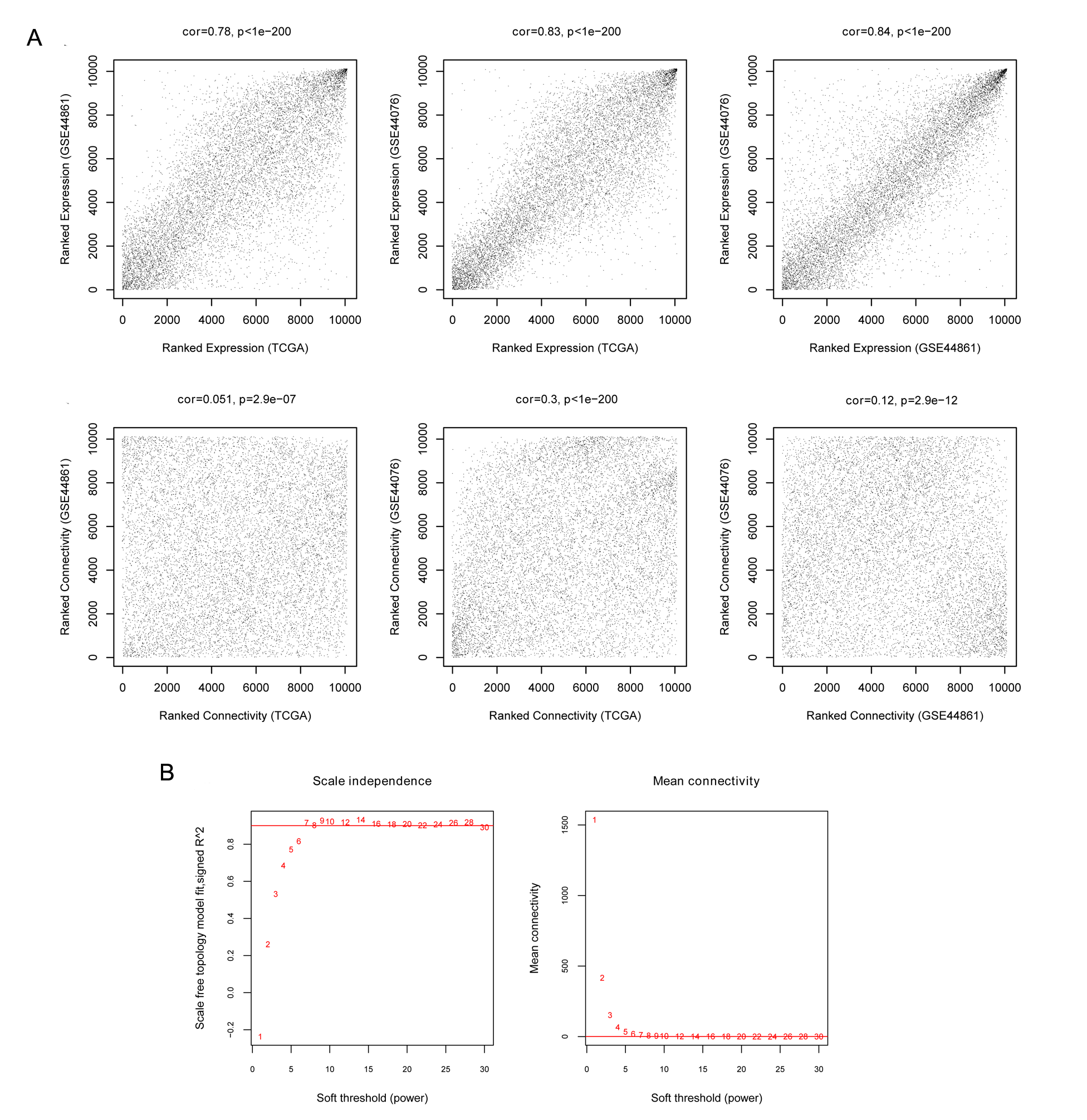

Supplement: Supplementary file 1 — Additional file 1: Figure S1. Weighed gene co-expression network analysis in the training (TCGA) and validation (GSE44861 and GSE44076) datasets. (A) The correlation between the gene expression profiles in the TCGA, GSE44861 and GSE44076 datasets. Upper: the correlation analysis of gene expression level in the training and validation datasets; Lower: the analysis of node connection in the training and validation datasets. (B) Scale independence of the weighted gene co-expression network analysis (WGCNA). Left: the diagram for selecting the soft threshold power: The x axis represents the power of the weighting parameter and the y axis represents the scale-free topology model fit signed R2 in the network; Right: The diagram of gene connectivity corresponds to power value. The red line indicates the value under different power parameter and the average node connectivity of 1. [file 12957_2020_2116_MOESM1_ESM.tif]
