# Supplementary material for: Identification of a prognostic gene signature of colon cancer using integrated bioinformatics analysis
Source: World J Surg Oncol. 2021 Jan 13;19:13. doi: 10.1186/s12957-020-02116-y (PMC7807455; doi:10.1186/s12957-020-02116-y)
Supplement: Supplementary file 2 — Additional file 2: Table S1. The list of the prognosis-associated differentially expressed genes across the three datasets (TCGA, GSE44861, and GSE44076) using the Cox regression analysis. [file 12957_2020_2116_MOESM2_ESM.docx]

Table S1. The list of the prognosis-associated differentially expressed genes across the three datasets (TCGA, GSE44861, and GSE44076) using the Cox regression analysis.

| **univariate Cox regression analysis** | | | |  |
| --- | --- | --- | --- | --- |
| **Gene** | **coef** | **Hazard Ratio** | **p** | |
| NAT1 | -0.665 | 0.514 | 4.55E-05 | |
| PCSK2 | 1.31 | 3.7 | 3.60E-04 | |
| TRPV4 | 0.602 | 1.83 | 6.00E-04 | |
| MFNG | 0.375 | 1.46 | 2.00E-03 | |
| CLCA1 | -0.107 | 0.899 | 2.05E-03 | |
| WDR78 | -1.1 | 0.334 | 2.05E-03 | |
| PPARGC1A | -0.545 | 0.58 | 2.10E-03 | |
| NAT2 | -0.291 | 0.748 | 2.20E-03 | |
| SLC4A4 | -0.376 | 0.687 | 2.20E-03 | |
| GPRASP1 | 0.575 | 1.78 | 2.35E-03 | |
| SULT1B1 | -0.235 | 0.791 | 2.75E-03 | |
| FCRL2 | 0.902 | 2.47 | 2.90E-03 | |
| POU3F4 | 5.65 | 284 | 2.90E-03 | |
| CLCA4 | -0.186 | 0.83 | 3.85E-03 | |
| CD72 | 0.551 | 1.74 | 4.30E-03 | |
| ZG16 | -0.135 | 0.874 | 4.60E-03 | |
| AFF3 | 1.23 | 3.42 | 4.95E-03 | |
| SCGB2A1 | -0.363 | 0.696 | 5.50E-03 | |
| PLCG2 | 0.524 | 1.69 | 6.50E-03 | |
| RUNX3 | 0.264 | 1.3 | 7.50E-03 | |
| NOTCH4 | 0.484 | 1.62 | 8.00E-03 | |
| TPSG1 | -0.202 | 0.817 | 8.00E-03 | |
| NOTCH3 | 0.269 | 1.31 | 8.50E-03 | |
| CPA3 | -0.211 | 0.81 | 9.50E-03 | |
| UGT2A3 | -0.191 | 0.826 | 9.50E-03 | |
| CD177 | -0.194 | 0.823 | 1.00E-02 | |
| FLT1 | 0.385 | 1.47 | 1.10E-02 | |
| PTPN14 | 0.491 | 1.63 | 1.15E-02 | |
| RASGRP2 | 0.505 | 1.66 | 1.15E-02 | |
| NLRP1 | 0.371 | 1.45 | 1.20E-02 | |
| AKAP5 | -0.61 | 0.543 | 1.30E-02 | |
| ARL4C | 0.223 | 1.25 | 1.30E-02 | |
| SP140 | 0.421 | 1.52 | 1.60E-02 | |
| ALDH1A3 | 0.3 | 1.35 | 1.70E-02 | |
| PRRX2 | 0.231 | 1.26 | 1.70E-02 | |
| SIGLEC1 | 0.299 | 1.35 | 1.70E-02 | |
| SEMA3E | 0.614 | 1.85 | 1.75E-02 | |
| FAS | -0.273 | 0.761 | 1.95E-02 | |
| FCGBP | -0.0901 | 0.914 | 1.95E-02 | |
| HSD17B14 | 0.309 | 1.36 | 1.95E-02 | |
| PCSK5 | 0.39 | 1.48 | 1.95E-02 | |
| A4GALT | 0.238 | 1.27 | 2.00E-02 | |
| SPHK1 | 0.203 | 1.22 | 2.10E-02 | |
| CA2 | -0.122 | 0.885 | 2.25E-02 | |
| DNASE1L3 | -0.329 | 0.719 | 2.25E-02 | |
| CCDC68 | -0.321 | 0.725 | 2.30E-02 | |
| CPM | -0.291 | 0.747 | 2.30E-02 | |
| LRRC19 | -0.176 | 0.838 | 2.30E-02 | |
| NUAK1 | 0.294 | 1.34 | 2.35E-02 | |
| ATP1B2 | 0.567 | 1.76 | 2.40E-02 | |
| CLDN8 | -0.409 | 0.664 | 2.40E-02 | |
| SERPINE1 | 0.149 | 1.16 | 2.40E-02 | |
| CA4 | -0.131 | 0.877 | 2.45E-02 | |
| TGFB3 | 0.196 | 1.22 | 2.55E-02 | |
| CBX7 | 0.37 | 1.45 | 2.60E-02 | |
| COMP | 0.114 | 1.12 | 2.60E-02 | |
| KCNJ8 | 0.295 | 1.34 | 2.65E-02 | |
| TEX11 | -1.91 | 0.148 | 2.70E-02 | |
| LAG3 | 0.264 | 1.3 | 2.90E-02 | |
| NOX4 | 0.432 | 1.54 | 3.00E-02 | |
| LGALS2 | -0.141 | 0.868 | 3.10E-02 | |
| SMPDL3A | -0.249 | 0.78 | 3.10E-02 | |
| NR3C2 | -0.252 | 0.777 | 3.15E-02 | |
| GUCA2A | -0.0964 | 0.908 | 3.20E-02 | |
| CNNM2 | 0.758 | 2.13 | 3.25E-02 | |
| ADORA3 | 0.451 | 1.57 | 3.40E-02 | |
| GUCA2B | -0.171 | 0.843 | 3.40E-02 | |
| TNFRSF17 | -0.248 | 0.78 | 3.45E-02 | |
| AOC3 | 0.151 | 1.16 | 3.50E-02 | |
| ASPA | 1.22 | 3.4 | 3.65E-02 | |
| BMP5 | -0.439 | 0.644 | 3.70E-02 | |
| FEV | 0.646 | 1.91 | 3.80E-02 | |
| MS4A12 | -0.127 | 0.881 | 3.80E-02 | |
| SRPX | 0.201 | 1.22 | 4.05E-02 | |
| RASGRP3 | 0.434 | 1.54 | 4.20E-02 | |
| BEST2 | -0.228 | 0.796 | 4.25E-02 | |
| C7 | 0.15 | 1.16 | 4.25E-02 | |
| EDN3 | -0.186 | 0.83 | 4.40E-02 | |
| MYOM1 | 0.39 | 1.48 | 4.45E-02 | |
| SPOCK1 | 0.154 | 1.17 | 4.55E-02 | |
| STAB1 | 0.2 | 1.22 | 4.55E-02 | |
| XDH | -0.177 | 0.838 | 4.60E-02 | |
| SPP1 | 0.0794 | 1.08 | 4.65E-02 | |
| FN1 | 0.0997 | 1.1 | 4.75E-02 | |
| multivariate Cox regression analysis | | | | |
| **Gene** | **coef** | **Hazard Ratio** | **P** | |
| NAT1 | -0.159476 | 0.8526 | 1.100E-04 | |
| WDR78 | -0.13201 | 0.8763 | 2.875E-02 | |
| TNFRSF17 | -0.121795 | 0.8853 | 5.810E-04 | |
| CPA3 | -0.098821 | 0.9059 | 2.930E-05 | |
| CPM | -0.085541 | 0.918 | 4.435E-03 | |
| PPARGC1A | -0.08452 | 0.919 | 5.571E-03 | |
| EDN3 | -0.044987 | 0.956 | 3.533E-02 | |
| FCGBP | 0.030908 | 1.0314 | 1.035E-02 | |
| PRRX2 | 0.05242 | 1.0538 | 3.940E-02 | |
| MFNG | 0.072883 | 1.0756 | 2.818E-03 | |
| PCSK5 | 0.074117 | 1.0769 | 3.477E-02 | |
| FCRL2 | 0.181645 | 1.1992 | 2.106E-02 | |
| ADORA3 | 0.223931 | 1.251 | 4.000E-04 | |
| ASPA | 0.435201 | 1.5453 | 3.542E-02 | |
